# The Prevalence, Incidence and Natural Course of Positive Antithyroperoxidase Antibodies in a Population-Based Study: Tehran Thyroid Study

**DOI:** 10.1371/journal.pone.0169283

**Published:** 2017-01-04

**Authors:** Atieh Amouzegar, Safoora Gharibzadeh, Elham Kazemian, Ladan Mehran, Maryam Tohidi, Fereidoun Azizi

**Affiliations:** 1 Endocrine Research Center, Research Institute for Endocrine Sciences, Shahid Beheshti University of Medical Sciences, Tehran, I. R. Iran; 2 Department of Epidemiology and Biostatistics, School of Public Health, Tehran University of Medical Sciences, Tehran, I.R. Iran; 3 Prevention of Metabolic Disorders Research Center, Research Institute for Endocrine Sciences, Shahid Beheshti University of Medical Sciences, Tehran, I. R. Iran; Hospital Universitari i Politecnic La Fe, SPAIN

## Abstract

**Objective:**

Thyroid peroxidase antibody (TPOAb), the most common antibody frequently measured in population surveys is a protein expressed in the thyroid gland. We conducted the present study to analyze the prevalence and incidence of thyroid auto immunity and natural course of TPOAb in a population based study.

**Material and Methods:**

This prospective study was conducted within the framework of the Tehran Thyroid Study (TTS) on 5783 (2376 men and 3407 women) individuals aged ≥ 20 years who had thyroid function tests at baseline and were followed up for median 9.1 year with TPOAb measurements at approximately every 3 years.

**Results:**

The mean age of total population at baseline was 40.04±14.32. At baseline, of the 5783 participants, 742 (12.8%) were TPOAb positive, with higher prevalence among women than in men (16.0 vs. 8.5%, p = 0.001). The prevalence of TPOAb positivity in the total population was 11.9, 14.9 and 13.6% in the young, middle age and elderly respectively. The total incidence rate (95%CI) of TPOAb positivity in the total population (5020) was 7.1 (6.36–7.98) per 1000 person-years of follow-up, with higher incidence of TPOAb positivity among young participants, i.e. 8.5 (7.5–9.7) per 1000 person-years. Sex specific incidence rate demonstrated that TPOAb positivity was higher in women, 9.3 (8.2–10.7) per 1000 person-years. The Cox's proportional hazard model analysis showed that the hazard ratio of developing TPOAb positivity was higher in women than men (P<0.0001) and tended to increase slightly with serum TSH levels (P<0.0001) but declined with increasing age (P<0.0001) in the total population. Our findings demonstrate that individuals, who became TPOAb positive in each phase, had significant elevation of TSH levels at the phase of seroconversion, compared to baseline values.

**Conclusion:**

Gender, age and elevated serum TSH were found to be risk factors for developing TPOAb positivity. Furthermore, compared to baseline a significant elevation of TSH levels during seroconversion phase was observed in TPOAb positive individuals.

## Introduction

Autoimmune thyroid disorders (ATDs) are the commonest autoimmune endocrine diseases, characterized by the frequent presence of auto antibodies directed against thyroglobulin (Tg), thyroperoxidase (TPO) and thyrotropin receptor (TRAB)[1,2]. The most common antibodies frequently measured in serum in population surveys are the thyroid peroxidase antibody (TPOAb) and thyroglobulin antibody (TgAb) [3]. TPOAb is a membrane-bound protein which is expressed in the thyroid gland and participates in catalyzing thyroid hormone synthesis at the apical membrane of the follicular cells[3].

Several studies report that antibody-dependent cell-mediated cytotoxicity could be induced by TPOAb[3,4]. Actually, TPOAb has frequently been found in the general population, compared to other antibodies; this antibody directly involved in thyroid cells damage and positively correlated with the activity of chronic autoimmune thyroiditis [5]. Thyroid autoimmunity could be initiated by genetic, environmental and endogenous factors[6]. The prevalence of thyroid antibodies has been measured in several studies and various TPOAb positive rates have been reported in different areas of the world [3,7–11], e.g. the National Health and Nutrition Examination Survey III (NHANES) reported that over 10% of adults were TPOAb or TgAb positive, with a prevalence of 13% for TPOAb and 11.5% for TgAb [7]; Pedersen et al also documented a prevalence rate of 13.1% for TPOAb in Danish population [3]. There are limited longitudinal studies investigating the incidence of thyroid antibodies; in the 20-year Whickham survey, 17% of women and 7% of men developed anti-thyroid antibodies [12]. Also, in a study by Li et al the 5-yr cumulative incidence of TPOAb-positivity was reported to be 2.81%[4]. These differences may be due to genetic, environmental factors (such as iodine intake) and methods applied for antibody measurement [13,14]. Several mechanisms have been postulated for the development of circulating TPOAb and TgAb. Some mechanisms focus on immune system disorders while others suggests abnormalities observed in presentation or structure of individual antigens [15]. Though the prevalence of TPOAb positivity has been investigated in several cross sectional studies, limited longitudinal studies have demonstrated the incidence and natural course of this antibody in the general population. We conducted the present study to analyze the prevalence, incidence rate, and natural courses of TPOAb in a population based study.

## Materials and Methods

### Study Population

The present study was conducted within the framework of the Tehran Thyroid Study (TTS), a prospective population-based cohort study performed on residents of district-13 of Tehran with the aim of evaluating the prevalence and natural course of thyroid diseases and their long term consequences in terms of metabolic and ischemic heart disease, cardiovascular and all-cause mortality in the urban, iodine sufficient population of Tehran, the capital of Iran. The TTS is a cohort study, being conducted within the framework of the Tehran Lipid and Glucose Study (TLGS). The TLGS study is an ongoing integrated community-based, with follow-ups at 3 year intervals, survey initiated in 1997 for the identification and prevention of non-communicable disorders (NCD).

For the TLGS initially, a total of 15,005 individuals, aged≥ 3 years, under coverage of 3 medical health centers in Tehran, were selected by multistage stratified cluster sampling; among these 10,368 participants were aged ≥20 years; 5783 of these subjects were selected to participate in the Tehran Thyroid Study. Details of the study design have previously been described[16].

At the first visit, the study was explained to subjects and demographic data were obtained. All clinical examinations were performed at the beginning of the study and collection of demographic, clinical and laboratory data was repeated every 3 years. Details of the study has been published elsewhere[16].

After obtaining the prevalence of TPOAb positivity in 5783 (2376 men and 3407 women, aged ≥ 20 years) TTS participants, we excluded 742 subjects who were TPOAb positive at baseline and 21 subjects lacking the data required on thyroid function tests (TFTs). To compute incidence rate, 5020 subjects, TPOAb negative at baseline, were followed. Furthermore, 38, 42 and 20 subjects who were pregnant at 1^st^, 2^nd^ and 3^rd^ phases of the study were also excluded.

### Laboratory measurements

Fasting blood samples were drawn from all participants between 7:00 and 9:00 AM into vacutainer tubes at each reassessment.

Free thyroxine (FT4) and thyroid stimulating hormone (TSH) were determined on -70°C stored serum samples by the electro chemiluminescence immunoasaay (ECLIA) method, using Roche Diagnostics kits & Roche/Hitachi Cobas e-411 analyzer (GmbH, Mannheim, Germany). Lyophilized quality control material (Lyphochek Immunoassay plus Control, Bio-Rad Laboratories) was used to monitor accuracy of assay; intra- and inter-assay CVs were 1.3% and 3.7% for FT4 and 1.5% and 4.5% for TSH determinations respectively. Thyroid peroxidase antibody (TPOAb) was measured by immune-enzymometric assay (IEMA) using commercial kits (Monobind, Costa Mesa, CA, USA) and the Sunrise ELISA reader (Tecan Co., Salzburg, Austria); intra- and inter-assay CVs were 3.9% and 4.7%, respectively. All laboratory measurements were performed in the same laboratory by skilled laboratory technician.

The ethical committee of the Research Institute for Endocrine Sciences (RIES) of Shahid Beheshti University of Medical Sciences approved the protocol for this study. Written informed written consent was obtained from all subjects.

### Definitions and terms

TSH and FT4 reference ranges were defined based on the reference ranges of the present population[17].

Euthyroidism was defined as TSH levels with the reference range of 0.32–5.06 mIU/L (0.32≤TSH≤5.06), while not taking any thyroid medication or agent interfering with thyroid function test results. Other thyroid test results such as TSH>5.06 mIU/L and FT4 of 0.91–1.55 ng/dL, TSH>5.06 mIU/L and FT4<0.91ng/dL, TSH<0.32mIU/L and FT4 between 0.91–1.55ng/dL, TSH<0.32 mIU/L and FT4>1.55(ng/dL) were considered as subclinical hypothyroidism, overt hypothyroidism, subclinical hyperthyroidism and overt hyperthyroidism, respectively[17].

Those with TPOAb levels > 40 IU/mL were regarded as TPOAb-positive and this cut off value was used to assess the prevalence and incidence of TPOAb positivity throughout the study. Since populations and assay-specific reference values for TPOAb have not been established for Iranian population yet, the upper 95% (+2SD) level of TPOAb (40 IU/mL) determined by the manufacturer in a normal population was used.

### Statistical analysis

Comparisons between subjects with and without TPOAb positivity were performed using the independent t-test (Mann-Whitney U for skewed) or chi-square tests.

We categorized age groups as follows: Young (20–45 y), middle-aged (46–65 y) and older (> 65 y).

Participants’ data were split according to gender. Cox proportional hazards models were used to evaluate associations of potential risk factors with incidence of autoimmunity in men and women separately. We compare incidence rate ratio and incidence risk difference for men and women in different age groups using 95% CI (results not shown).

The event date for autoimmune cases was described as the middle-time between the date of follow-up visit at which TPOAb reaches the threshold of 40 for the first time, and that of the most recent follow-up visit preceding the diagnosis. The follow-up time was drawn from the difference between the calculated mid-time date and the date at which the subjects entered the study. For the censored or lost to follow-up subjects, the survival time was the interval between the first and last observation dates. Follow-up duration and person-years were calculated using the measured survival time. To evaluate risk of thyroid dysfunction and associated factors in each phase of study, we used multinomial logistic regression (Euthyroid was considered as reference category). All analyses were performed using IBM SPSS for Windows version 21.0 and STATA version 12 SE (StataCorp LP, TX, USA), with two-tailed P values< 0.05 being considered significant.

## Results

At baseline, of 5783 participants, 742(12.8%) were TPOAb positive. Baseline characteristics of study participants with and without TPOAb positivity are illustrated in Table 1. The mean age of the total population at baseline was 40.04±14.32 and 2171(43.4%) were men. Those with TPOAb positive levels were older, more likely to be women (72.9%) and had higher levels of serum TSH, compared to the TPOAb negative group; 3.2 (1.6–6.1) mU/L vs 1.5(0.9–2.3) mU/L, P<0.01, respectively. Median TPOAb levels of individuals with positive and negative TPOAb were 177(79–366) mU/L and 5 (3–8) mU/L respectively. In the TPOAb positive group, 462(62.3%), 81(10.9%) and 25(3.4%) were euthyroid, overt hypothyroid and hyperthyroid, respectively. The prevalence of TPOAb positivity was significantly higher among women than in men (16.0 vs. 8.5%, p = 0.001). In the total population, prevalence of TPOAb positivity was 11.9%, 14.9% and 13.6% in young, middle age and elderly subjects, respectively. The prevalence of TPOAb positivity increased with age in women, being 14.8, 17.9 and 22.0% in young, middle age and elderly subjects, respectively).

**Table 1:** Baseline characteristics of study participants based on presence of thyroid autoimmune disease, Tehran Thyroid Study, 1999–2011.

Baseline	TPOAb positive (n = 742)	TPOAb negative (n = 5020)	P-value
Age(years)	42±14	40±14	<0.01
Sex %			<0.01
• Women	541(73.0)	2849(56.72)	
• Men	201(27.0)	2171(43.24)	
FT4(ng/dl)	1.18±0.93	1.24±0.24	0.13
TSH(mU/L)	3.25(1.65–6.17)	1.5(0.91–2.36)	<0.01
TPOAb(mU/L)	177(79–366)	5(3–8)	<0.01
Smoker %	49(6.06)	401(7.98)	0.31

TSH: Thyroid stimulating hormone; FT4: Free thyroxine; TPOAb: Thyroid Peroxidase Antibody

Values with normal distribution are presented as mean±SD; values with abnormal distribution are presented as median (Q1, Q3) and categorical variables are presented as (%).

Table 2 shows thyroid autoimmunity incidence per 1000 person-years, based on age and gender. During a median follow-up of 9.1 years, of 5020 eligible participants (2171 men and 2849 women) of the TTS (contributing to a total of 42098 person-years follow-up), 300 new cases of TPOAb positivity were diagnosed, including 223(74.3%) women and 77(25.7%) men. The incidence rate (95%CI) of TPOAb positivity in the total population was 7.1 (6.36–7.98) per 1000 person-years of follow-up, with higher incidence of TPOAb positivity among young participants 8.5 (7.5–9.7) per 1000 person-years. Sex specific incidence rate demonstrated that conversion to TPOAb positivity was higher in women, compared to men: 9.3(8.2–10.7) vs 4.2(3.4–5.3) per 1000 person-years respectively; the highest incidence rate of TPOAb positivity was observed in young women (10.7 per 1000 person-years).

**Table 2:** Thyroid autoimmunity incidence per 1000 Person-Years by age and gender, Tehran Thyroid Study, 1999–2011.

	No. of Participants	Person-Years of Follow-up	Incident Cases	Incidence Rate (95%CI) per 1000 Person-Years ^¶^
**Total Population**				
Young	3344	27659	236^†^	8.53(7.51–9.69)
Middle-Aged	1396	12065	55	4.55(3.50–5.93)
Elderly	280	2327	9	3.79 (1.97–7.29)
Total	5020	42097	300	7.12(6.36–7.98)
**Women**				
Young	1950	16093	173	10.74 (9.26–12.47)
Middle-Aged	796	6848	46	6.71 (5.03–8.96)
Elderly	103	905	4	4.41(1.65–11.77)
Total	2849	23847	223*	9.35 (8.20–10.66)
**Men**				
Young	1394	11565	63	5.44 (4.25–6.97)
Middle-Aged	600	5217	9	1.72 (0.89–3.13)
Elderly	177	1467	5	3.40 (1.41–8.18)
Total	2171	18249	77*	4.21(3.37–5.27)

^¶^95% Confidence intervals (CI) were calculated using Fisher’s exact test.

*The incidence rate of TPOAb positivity was significantly higher in women than men, p<0.05

† Incidence of TPOAb positivity was higher in young individuals compared to middle-aged and elderly, p<0.05

According to incidence rate difference (IRD) and incidence rate ratio (IRR) indices, differences between men and women in the total population and age groups were statistically significant.

The Cox proportional hazard analysis for predicting development of TPOAb positivity based on potential confounding effects of age at baseline, gender, and baseline serum FT4 and TSH values are depicted in Table 3. The analysis showed that the hazard ratio of developing TPOAb positivity was higher in women than in men (P<0.0001) and tended to rise slightly with increasing serum TSH levels (P<0.0001) but decreased with increasing age (P<0.0001) in the total population.

**Table 3:** Results of Cox regression analysis of the development of TPOAb positivity by individual baseline variables.

Variables	HR	95% CI	Standard Error	*P*
**Total population**				
Sex†	2.06	1.58–2.68	0.27	< .0001
Age(years)	0.97	0.97–0.98	0.004	< .0001
TSH(mU/L)	1.01	1.01–1.01	0.002	< .0001
**Women**				
Age(years)	0.98	0.97–0.99	0.005	< .0001
TSH(mU/L)	1.01	1.00–1.01	0.002	< .0001
**Men**				
Age(years)	0.98	0.96–0.99	0.008	0.02
TSH(mU/L)	1.01	0.98–1.05	0.019	0.37

HR: Hazard ratio; TSH: Thyroid stimulating hormone; FT4: Free thyroxine; TPOAb: Thyroid peroxidase antibody

†Male sex was considered as reference.

Serum TSH and FT4 changes in those who became TPOAb positive in each phase are shown in Table 4; 116, 117 and 67 subjects seroconverted after 3,6, and 9 years follow up respectively. Study results indicate that individuals, who became TPOAb positive in each phase, had significantly elevated TSH levels during the seroconversion phase, compared to their baseline values. Furthermore, seroconverted TPOAb positive subjects at their 6 year follow up had only an elevated serum FT4, compared to their baseline levels (1.58 vs. 1.26 p = 0.01); although this increase was not clinically important.

**Table 4:** Serum FT4 and TSH concentration in participants who had seroconverted at 3, 6, and 9 yr follow up assessments.

	Seroconversion										
	After 3 years follow up (n = 116)			After 6 years follow up (n = 117)				After 9 years follow up (n = 67)			
	Baseline	3 years follow up	p-value	baseline	6 years follow up	P-value		baseline		9 years follow up	P-value
TSH(mU/L)	2.13(1.44–3.61)	2.57(1.46–4.35)	<0.01	2.29(1.26–3.81)	3.19(1.79–6.04)		<0.01		2.06(1.24–3.49)	3.07(1.88–6.78)	<0.01
FT4(ng/dl)	1.15±0.22	1.18±0.30	0.16	1.26±0.41	1.58±0.24		0.01		1.20±0.19	1.36±2.31	0.56

TSH: Thyroid stimulating hormone; FT4: Free thyroxine;Values with normal distribution are presented as mean±SD; Values with abnormal distribution are presented as median (Q1, Q3). 116, 117 and 67 subjects seroconverted after 3, 6, and 9 years follow up respectively.

Risk analysis of developing hypothyroidism and hyperthyroidism among TPOAb positive subjects are depicted in Table 5. We observed that TPOAb positive subjects had a higher risk of developing hypothyroidism in all phases; after 3 years: relative risk [RR] 4.14, 95% confidence interval [CI] 2.57–6.67; after 6 years: relative risk [RR] 7.57, 95% confidence interval [CI] 5.30–10.82; and after 9 years: relative risk [RR] 4.21, 95% confidence interval [CI] 2.35–7.55. Furthermore, results indicated that TPOAb positive individuals were approximately 2.5 times more likely to develop hyperthyroidism after 6 years of follow up (relative risk [RR]; 2.35; 95% confidence interval [CI]; 1.11–4.99).

**Table 5:** Risk analysis of developing hypothyroidism and hyperthyroidism among TPOAb positive subjects.

Years of follow up	Hypothyroidism		Hyperthyroidism	
	RR	95% CI	RR	95% CI
3 yr	4.14	2.57–6.67	2.35	1.11–4.99
6 yr	7.57	5.30–10.82	2.27	0.89–5.78
9 yr	4.21	2.35–7.55	2.62	0.60–11.45

RR; Relative risk

**Fig 1:**
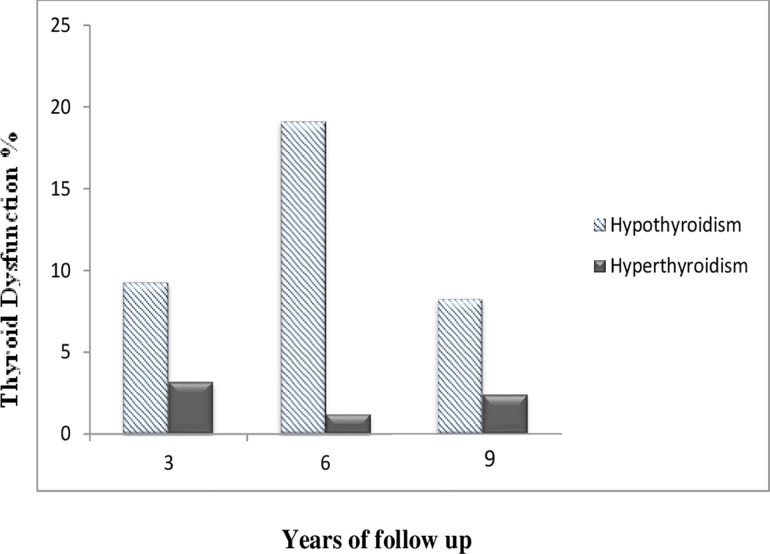
The incidence rate of hypothyroidism and hyperthyroidism in euthyroid TPOAb positive subjects in each phase.

To study the trend of thyroid dysfunction in euthyroid subjects who became TPOAb positive during the study, we calculated the incidence proportion of hypothyroidism and hyperthyroidism in this group in each phase (Fig 1). After 3 years follow up, among those who were euthyroid and TPOAb positive (n = 248), 162 subjects (65.3%) remained euthyroid, whereas 23(9.3%) and 8(3.2%) progressed to subclinical and overt hypothyroidism and hyperthyroidism respectively. Likewise, among TPOAb positive euthyroid individuals (n = 162) after 3 years follow up, 117(72.2%) remained euthyroid whereas 31(19.1%) and 2(1.2%) developed subclinical and overt hypothyroidism and hyperthyroidism respectively after 6 years follow up. Finally, of 84 TPOAb positive euthyroid individuals who were euthyroid after 6 years follow up, 75(89.3%) remained euthyroid while 7(8.3%) and 2(2.4%) developed subclinical hypothyroidism and subclinical and overt hyperthyroidism respectively after 9 years follow up.

## Discussion

In the current study, we found positive TPOAb at baseline in 8.5% of men and 16.0% of women in the total population and the crude incidence rate of TPOAb positivity was 7.1 per 1000 person-years of follow-up. Findings also demonstrate that female sex, elevated serum TSH value and having younger age at baseline were predictive of development of TPOAb positivity during the study.

In the HUNT study, positive TPOAb was found in 2.8% of men and13.9% of women, aged over 40 y of age [8]. In another study conducted in Denmark, TPOAb titers >200 kU/l was reported in 16.9% of women and 6.6% of men [9]. In NHANES III, positive TPOAb were documented in 8.7% of men and 17.0% of women [7] and a study by Hoogendoorn et al also reported a positive TPOAb status in 8.6% of men and 18.5% of women[10]. Furthermore, the results of the current investigation revealed that subjects with TPOAb positive status at baseline were older, more frequently women and had higher levels of serum TSH, compared to the TPOAb negative group. The higher prevalence of positive TPOAb in women than men has previously been well documented which is consistent with the higher prevalence of AITD in women [3,18–20]. In the present study, the prevalence rate of TPOAb positivity in the total population increased with age until the age range of 46–65 years, after which a slight decrease in the prevalence rate of TPOAb was observed; however in women, the prevalence rates increased with age continuously. Preceding reports have been shown that the prevalence rate of thyroid antibodies increases until certain age and after that it either remains unchanged or even decreases sometimes [3,21]; this age-dependency however has not been reported for men in all studies [20,22,23] which is in agreement with the results of our study. However, one study did not report an increased prevalence rate of positive TPOAb with age [8].

Indeed, the prevalence of positive TPOAb reported by different epidemiological studies is difficult to compare because different biochemical assays and epidemiological methods are often applied and no internationally standard assays are available [3,8]. Thyroid antibody assays vary greatly in technical detail and cut-off values used [3,8]. Furthermore, the sensitivity and specificity of the different types of antibody assays to measure thyroid antibodies vary [3,8]. In addition, the age and sex distribution of various studies often differ widely, and results from these studies are difficult to compare.

The results of current study showed that the crude incidence rate of TPOAb positivity was 7.1 per 1000 person-years of follow-up in the total population, with higher incidence of TPOAb positivity, among women, who were younger at baseline. Additionally, the sex specific incidence rate demonstrated that, compared to men, the incidence of TPOAb positivity was higher in women. Only few studies have investigated the incidence of thyroid autoantibodies in the general population. In the 20-year Whickham survey, 17% of women and 7% of men developed anti-thyroid antibodies since the first visit[12]; the study also showed age distribution of anti-thyroid antibodies only in women; the peak incidence of anti-thyroid antibodies was observed in the group aged 55–65 years at follow-up, i.e. age 35–45 years at first visit, which was consistent with the results of our study[12]. In another study conducted by Li et al in a Chinese population with different iodine intakes, the 5-yr cumulative incidence of TPOAb-positive was 2.81%, with no differences between men and women [4]; these differences in results might be due to the short interval between the first survey and follow-up of their study as much more time was needed for development of positive TPOAb [4]. The role of environmental factors e.g. Iodine intake and genetic determinants in the development of thyroid disorder has been suggested by previous studies[8,24]. Evidence on the contribution of genetic factors in the development of autoimmune thyroid disease has been demonstrated by studies conducted within families and in twins[8,25] and some genes or loci are believed to be involved in the development of autoimmune thyroid disease [25,26]; obviously, the studies differ in design, study population, participants' iodine intake, definition of diseases, and laboratory assays, all of which make it difficult to ascertain whether the differences actually exist.

Notably in the current study, TPOAb was measured in the general population from an iodine sufficient area. Iran was severely iodine deficient about two decades ago, following implementation of universal salt iodization however initiated in 1994, Iran was declared to be IDD free by the WHO/UNICEF. Adequate median urinary iodine with over 95% of households’ consumption of iodized salt was reported and confirmed by ongoing monitoring national surveys in 1996, 2000 and 2006.[27]

Our study demonstrated that sex and age were risk factors for developing TPOAb positivity, findings not in agreement with those of Li et al who did not find age and gender to be risk factors for developing TPOAb positivity [4]; one explanation for this discrepancy may be both the higher percentage of women and lower percentage of participants, aged over 55 y in this study, which reduced the study power needed to detect this relation. Also, we observed that each unit increase in TSH value was accompanied by a 1% increase in TPOAb positivity, although this observation was statistically significant, it was not clinically important, which may be due to a large sample size, may explain resulting this significant association. Also as observed TPOAb positivity was accompanied with increased risk of hypothyroidism, the relationship may be as a result of pathologic changes in the thyroid gland which occurs long before manifestation of TPOAb positivity status.

The results of current study indicate that individuals, who became TPOAb positive had elevated TSH levels during the seroconversion phase, compared to their baseline values. Also, we observed that being TPOAb positive was accompanied with higher risk of developing hypo- and hyperthyroidism. Notably, the results of the present study are in agreement with those of previous studies; the Whickham and Busselton cohort study reported that increasing levels of serum TSH, accompanied with TPOAb positivity status, could predict the incidence of overt hypothyroidism[12,28]. Bjoro et al reported that the TSH values were higher in patients with positive TPOAb, compared to subjects with negative antibodies. Moreover, higher percentage of elevated TSH was reported in TPOAb positive individuals, compared to the TPOAb negative group, strongly indicating that TPOAb appearance in the circulation was observed long before the change of thyroid function demonstrated by changes in TSH values[8]. Our results were also in agreement with those of Strieder et al who reported TPO antibody concentration above 100 kU/L to be an independent risk factor for developing thyroid dysfunction[29].

In a longitudinal study, Li et al demonstrated that positive thyroid antibodies are associated with developing hypothyroidism; their results revealed that euthyroid individuals with positive antibodies at baseline progressed to elevated TSH levels more often than in those with negative antibody status after a 5 year follow up; their study demonstrated that TPOAb positivity could increase the incidence of developing thyroid dysfunction and it should be considered as a risk factor for thyroid dysfunction in particular, hypothyroidism[4]. Likewise, Tunbridge et al reported elevated TSH values and positive thyroid antibodies to be associated with development of overt hypothyroidism, after 4 years follow up[30]. Knudsen et al showed that the TPOAb titres >200 kU/l were found to be higher in participants with TSH >5 mU/l than in individuals with normal TSH values, suggesting autoimmune thyroiditis to be the primary cause of hypothyroidism[9]. Furthermore, in a population-based survey conducted in the Netherlands, TPOAb positivity was associated both with abnormally increased and decreased TSH concentrations[10].

The current study has several limitations which should be addressed; first we did not measure TgAb. Second, although detailed history was obtained at the time of the examination, use of thyroid or other medications and the presence of thyroid diseases such as goiter were self-reported. Although, urinary iodine excretion was not measured, This study has been conducted in an iodine sufficient country. Among the strengths of our study, our study is one of the few long term follow up studies which provides incidence data for TPOAb status over a median 9.1 yr follow-up in different age and sex groups, which also allowed us to determine the thyroid function of a population based on their TPOAb status. Furthermore, to the best of our knowledge, this is the first longitudinal study which assesses the risk factors for developing TPOAb positivity. Also for measurement of TPOAb, we used the immune-enzymometric assay(IEMA) which has higher sensitivity and specificity, compared to assays based on passive hemagglutination in detecting thyroid autoimmunity[3].

In conclusion, following assessment of TPOAb status in a general population with sufficient iodine intake, we found the age and sex dependent incidence rate for TPOAb positivity in the total population and observed that TPOAb positivity was associated with elevated TSH and development of thyroid dysfunction, in particular hypothyroidism. Likewise, female sex, age and a slight increase in serum TSH values were risk factors for developing TPOAb positivity.

## Supporting Information

S1 File Supporting Information file (minimal data set). We randomly selected 5% of our data set.
(SAV)
